# The Effect of Novel Laser-Activated Bleaching Protocols on the Color Change of Non-Vital Anterior Teeth: An *In Vitro* Study

**DOI:** 10.1055/s-0044-1795119

**Published:** 2024-12-30

**Authors:** Inas EL Zayat, Mohamed Bahgat Abdel Hamid, Ahmed Tarek Farouk, Hatem Mostafa El-Damanhoury

**Affiliations:** 1 Restorative Dentistry Department, Faculty of Oral and Dental Medicine, Misr International University, Cairo, Egypt; 2 AALZ, RWTH Aachen University, Aachen, Germany; 3 Department of Preventive and Restorative Dentistry, College of Dental Medicine, University of Sharjah, Sharjah, United Arab Emirates; 4 Research Institute for Medical & Health Sciences, University of Sharjah, Sharjah, United Arab Emirates

**Keywords:** diode laser, internal bleaching, walking-bleach, SEM, discoloration

## Abstract

**Objective:**

This study compares the color change of non-vital anterior teeth after laser-activated bleaching and conventional walking bleaching technique.

**Materials and Methods:**

Sixty extracted teeth were endodontically treated, stained in a black tea solution, and the baseline shade was measured using a spectrophotometer (Easyshade, VITA). Bleaching was done using either: internal bleaching with 35% H _2_ O _2_ (Opalescence Endo) and then tooth sealed for 5 days (Gr1), 35% H _2_ O _2_ (JW Next) for 7 minutes (Gr2), internal and external bleaching for 7 minutes (Gr3), diode laser-activated internal bleaching for 30 seconds (940 nm, continuous wave, 2 W, noncontact mode, 300 um, non-initiated tip), wait for 7 minutes, second laser application for 30 seconds, tooth sealed for 5 days (Gr4), diode laser-activated internal bleaching for 24 hours (Gr5), or diode laser-activated internal and external bleaching for 24 hours (Gr6) ( *n* = 10). The color change (ΔE _00_ ) was measured and data were analyzed using one-way analysis of variance followed by Tukey post hoc test ( *a* = 0.05). The inner dentin of the samples was inspected using scanning electron microscopy.

**Results:**

All the tested bleaching techniques were able to change the color. All the laser-activated bleaching protocols, namely, Gr4, Gr5, and Gr6, showed higher mean ΔE _00_ values than the non-laser-activated bleaching Gr2 and Gr3 ( *p* < 0.05) and were statistically similar ( *p* > 0.05) to the control group Gr1. Laser-activated bleaching caused surface modification and dentinal tubule opening.

**Conclusion:**

All the tested laser-activated bleaching protocols showed faster and more efficient color change, comparable to the conventional 5-day walking bleaching protocol.

## Introduction

Dental aesthetics in conjunction with minimally invasive approaches have become increasingly crucial when considering diagnosis and treatment planning. Patients seek highly aesthetic restorative treatments, especially when dealing with the smile zone. Non-vital tooth discoloration might have many reasons, including dental trauma, the presence of necrotic debris that exists on the pulp horns, poor irrigation protocols, or even endodontic sealer remnants located in the pulp chamber or approaching the chamber walls. 1 2

Among the most common causes of tooth discoloration is intracoronal blood decomposition, creating an aesthetic challenge for clinicians to restore the original tooth color. 3 The impact of this pigmented tooth is remarkable since such isolated chromatic change will show an obvious discrepancy with the surrounding teeth. 4 5 6 Internal tooth bleaching offers a minimally invasive and conservative solution with a much lower cost, in the treatment of discolored nonvital anterior teeth. Many internal bleaching techniques were described in the literature many years ago, proving their efficiency in the reversion of these chromatic changes. The walking bleach technique is considered among the most commonly used internal bleaching techniques. In this technique, the endodontic treatment is sealed with a glass ionomer barrier followed by the insertion of the bleaching gel into the chamber and sealing of the access cavity with a temporary filling material. 7 8 The applied bleaching agent is then refreshed weekly until it meets the patient's aesthetic demands by achieving color harmony with the adjacent teeth.

In-office bleaching protocols using hydrogen peroxides in high concentrations in combination with a light source could play an integral role in lightening these pigmented teeth. Power in-office bleaching employing a light source in the presence of added pigment in the bleaching gel will result in higher absorption of light by the gel. Lasers could be used as a powerful tool to accelerate the release of free radicals within the bleaching gel, decreasing the time needed to complete the whitening procedure. 9 Up to date there is no consensus on the best protocol for using laser-activated bleaching to obtain immediate successful results with internally discolored teeth. This study aims to evaluate the effect of the application of lasers-activated bleaching with different protocols on the color change of nonvital anterior teeth and compare it to the conventional walking bleaching technique. The null hypothesis to be tested was that the tested laser-activated and nonlaser-activated bleaching methods would not significantly affect the whitening of the teeth after staining.

## Materials and Methods

### Sample Size Calculation

The total sample size was determined using power analysis with an effect size ( *f* ) of 0.49, using alpha ( *α* ) level of 0.05 (5%) and beta ( *β* ) level of 0.20 (20%), that is, power = 80%; the minimum estimated sample size was a total of 60 subjects. Calculation was based upon the results of a previous study. 9 So, the total sample size was 60, and each group included 10 subjects. Sample size calculation was performed using G*Power Version 3.1.9.2.

### Sample Preparation

Sixty freshly extracted, decay-free human permanent maxillary incisors were used in this study. Teeth were obtained from the Oral Surgery Department of Misr International University after patient informed consent and following an approved protocol by the university research ethics committee with an IRB number MIU-IRB-2324-052. Prior to experimental use, the teeth were examined for the presence of cracks or other surface defects. The teeth were cleaned of any soft tissue covering the root surface using a curette and any hard deposits were cleaned with an ultrasonic device. Teeth were stored in distilled water at room temperature until they were immersed in the staining solutions.

A standard endodontic access cavity was prepared in each tooth by using a diamond bur, size 018 (6801, Komet Dental, Germany) in a high-speed handpiece. The pulps were extirpated, and the root canals were instrumented manually with #10, #15, and #20 K-files (M.acess, Dentsply Maillefer, Ballaigues, Switzerland). The canal instrumentation was accomplished using rotary files (2shape, Micro-Mega, Besancon, France), ending with size 25 with taper .06. Canal irrigation during cleaning and shaping was performed using 5.25% sodium hypochlorite (Cerkamed, Stalowa Wola, Poland) and normal saline. The root canals were dried with paper points and obturated using a single-cone technique using a resin-based endodontic sealer (AH Plus, Dentsply Sirona, Konstanz, Germany). Teeth were kept in distilled water for 1 week, then each tooth was mounted in a Teflon block and the root surface was immersed in a light-body silicon impression material (Hydrorise, Zhermack, Italy) to the level of the cementoenamel junction (CEJ) to simulate the gingival tissues and provide a hermetic seal of the root surface. The gutta-percha was removed from the orifice to 2 mm beneath the CEJ by using gates drills size 3 (Mani, Tochigi, Japan). Note that 2 mm of glass ionomer restorative material (KetacFil Plus Aplicap, 3M ESPE, St. Paul, Minnesota, United States) was placed as a base to seal the orifice of the root canal to prevent radicular leakage of the bleaching material.

A black tea solution was prepared by boiling 2 g of tea (Ahmad Tea, London, United Kingdom) in 100 mL of distilled water for 5 minutes and filtered through gauze to remove the tea from the infusion. Once the baseline shade of each of the specimens was detected, the samples were immersed in the tea solution at room temperature in tightly sealed plastic universal containers at 37°C. The staining solution was changed daily, and the color change was checked daily over 6 days after the specimen's insertion in the solution.

Teeth were divided randomly into six groups ( *n* = 10), according to the bleaching method:

Group 1 (Gr1): Application of 35% H _2_ O _2_ (Opalescence Endo, Ultradent, South Jordan, Utah, United States) internally and sealed with glass ionomer restoration for 5 days as a control group and then color change assessment was done.
Group 2 (Gr2): Application of 35% H _2_ O _2_ (JW Next, Heydent GmbH, Kaufering, Germany) internally for 7 minutes and then immediate color change assessment after 24 hours.
Group 3 (Gr3): Application of 35% H _2_ O _2_ (JW Next) internally and externally for 7 minutes and then immediate color change assessment after 24 hours.
Group 4 (Gr4): Application of 35% H _2_ O _2_ (JW Next) internally, followed by laser activation using diode laser 940 nm wavelength (EpicX, Biolase) in continuous wave with output power 2 W in noncontact (10 mm away) using 300 um non-initiated tip, applied for 30 seconds and then waited for 7 minutes to avoid heat deposition to the surrounding tissues, followed by second laser application for 30 seconds, after that sealed with a temporary restoration for 5 days and then color change assessment was done.
Group 5 (Gr5): Application of 35% H _2_ O _2_ (JW Next) internally followed by laser activation with the same protocol and then immediate color change assessment after 24 hours,
Group 6 (Gr6): Application of 35% H _2_ O _2_ (JW Next) internally and externally followed by laser activation following the previously mentioned protocol and then immediate color change assessment after 24 hours.

**Fig. 1:**
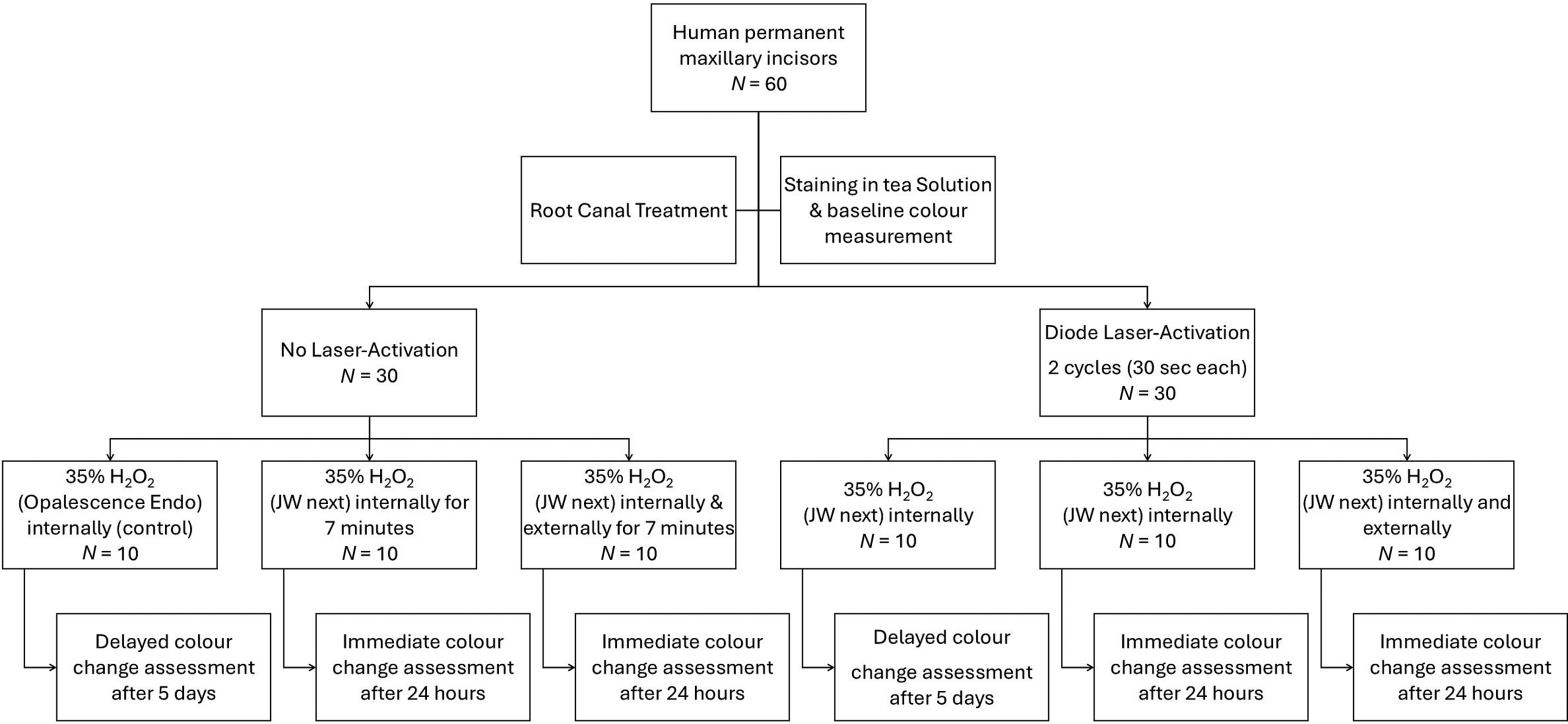
Flowchart of study design.

The study design is illustrated in Fig. 1 and the bleaching materials investigated and their corresponding active ingredients, contents, mixing procedure, and manufacturer information are listed in Table 1 .

**Table 1:** The bleaching materials investigated and their corresponding active ingredients, contents, mixing procedure, and manufacturer

Bleaching gel	Material type	Manufacturer	Active ingredient (w/w%)	Color	Mixing/Procedure
JW Next	Laser-activated bleaching	Heydent GmbH, Kaufering, Germany	H _2_ O _2_ (35%)	Red	Double-cartridge syringe/self-mixing tips
Opalescence Endo	Walking bleach	Ultradent, South Jordan, Utah, United States	H _2_ O _2_ (35%)	Dark red	Syringe-to-syringe mixing

### Color Change Assessment

An electronic shade detection device (Easyshade V, VITA Zahnfabrik, Bad Säckingen, Germany) was used in this study for color assessment. The device is an intraoral reflectance spectrophotometer consisting of a base unit and a handpiece. The unit provides a full-spectrum light source and fiber optic bundles. The device has the ability to prevent the unit from making a color reading if a motion or improper angulation is detected. The device was primarily calibrated once switched on, as recommended by the manufacturer, by placing the instrument in the calibration block holder. The calibration process was repeated after every 10 readings to ensure accurate and precise readings. A special holder with a circular window was fabricated from a transparent polyvinyl siloxane impression material (Exaclear, GC Corp., Tokyo, Japan) to standardize the point of measurement at the same spot in the middle third of the tooth to see whether there was a change in the tooth value.

The color measurement was recorded for each tooth at the baseline and repeated after each treatment. The color difference was calculated between the color coordinates before (baseline) and after aging treatment, by calculating the CIEDE2000 color difference according to the following equation 10 :





where Δ *L* ′, Δ *C* ′, and Δ *H* ′ are the differences in each parameter, respectively, lightness, chroma, and hue, for a pair of samples using CIEDE2000. The weighting functions ( *S _L_* , *S _C_* , and *S _H_* ) adjust the total color difference for variation in the location of the color difference pair in *L* ′, *C* ′, and *H* ′ coordinates. The parametric factors ( *K _L_* , *K _C_* , and *K _H_* ) are correction terms for experimental conditions. Finally, a rotation function ( *R _T_* ) accounts for the interaction between chroma and hue differences in the blue region.

### Scanning Electron Microscopy Micromorphological Analysis

Three representative samples from each group ( *n* = 3) were used for the dentin surface morphological analysis. The teeth were fractured longitudinally at the middle of the crown, by creating a shallow groove along the crown, and then a #15 scalpel blade and hummer were used to complete the fracture without disturbing or contaminating the dentin surface. Then, each half of the crown was dehydrated in a desiccator containing dehydrated silica gel at room temperature for 24 hours. The inner dentin surfaces subjected to the bleaching protocol were sputter-coated with 100 Å gold-palladium (EMS 7620 Mini Sputter Coater, Hatfield, Pennsylvania, United States). Microphotographs of the treated dentin surfaces were captured under different magnifications up to 5,000 × , using a scanning electron microscope (SEM) (VEGA3 XM – TESCAN, Kohoutovice, Czech Republic) operating at 10 kV acceleration voltage and 17 mm working distance.

### Statistical Analysis

The measured Δ *E* and Δ *L* data were analyzed using SPSS software (IBM SPSS Statistics V24.0, IBM Corp., New York, United States). A one-way analysis of variance was used to assess the effect of the bleaching protocol on the total color change. Tukey post hoc test was used to detect pairwise differences among experimental groups. A 95% confidence level was applied for all the statistical tests ( *a* = 0.05), and the power of the study was 0.90.

## Results

The results of the study are summarized in Table 2 . The results revealed that all the tested bleaching techniques were able to change the color. All the laser-activated bleaching protocols, namely, Gr4, Gr5, and Gr6, showed the highest mean Δ *E*_00_ values and were statistically similar ( *p* > 0.05) to the control group Gr1. In comparison, the nonlaser-activated bleaching Gr2 and Gr3 exhibited significantly lower mean Δ *E*_00_ values ( *p* < 0.05) compared to the laser-activated groups and the control.

**Table 2:** Mean ∆ *L* and Δ *E*_00_ values and standard deviation of the tested bleaching protocols

	Group 1	Group 2	Group 3	Group 4	Group 5	Group 6
∆ *L*	21.98 ± 3.02 ^A^	5.78 ± 0.96 ^C^	15.73 ± 1.47 ^B^	19.59 ± 1.76 ^A^	18.61 ± 3.51 ^A^	21.25 ± 2.50 ^A^
∆ *E*_00_	17.57 ± 2.46 ^A^	5.31 ± 0.34 ^C^	13.02 ± 1.29 ^B^	14.81 ± 1.46 ^A^	14.32 ± 2.48 ^A^	16.20 ± 1.93 ^A^

Note: Within a raw, values with different superscript letters are statistically significantly different ( *p* < 0.05).

**Fig. 2:**
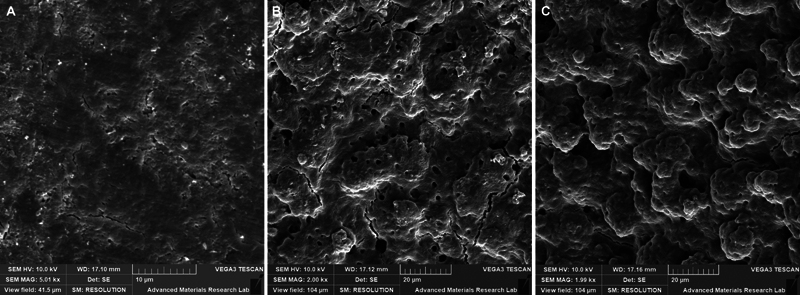
Representative scanning electron microscopy (SEM) microphotographs of ( **A** ) the untreated dentin surface completely covered with smear layer, ( **B** ) dentin surface after the application of 38% H _2_ O _2_ bleaching gel for 1 week, and ( **C** ) dentin surface treated with diode laser-activated bleaching with 38% H _2_ O _2_ showing complete removal of the smear layer and open dentinal tubules.

The SEM microphotographs are presented in Fig. 2A–C . The untreated dentin surface is shown in Fig. 2A , with the dentin surface covered with a thick smear layer completely obliterating the dentinal tubules. The use of the bleaching gel alone without laser activation did not significantly modify the dentin surface and no difference in dentin topography was found between the immediate (24 hours) and delayed (7 days) groups ( Fig. 2B ), as it appeared covered with a smear layer with partial opening of some dentinal tubules. The application of laser-activated bleaching, in both the immediate (24 hours) and delayed (7 days) groups, resulted in a similar substantial surface modification ( Fig. 2C ), where the dentin surface showed the absence of a smear layer, craters-like surface irregularities, and open dentinal tubules.

## Discussion

This study investigated a novel protocol for internal bleaching using diode laser-activated hydrogen peroxide. The results of the study showed that immediate laser-activated bleaching can achieve a comparable whitening effect to that achieved by 5 days of nonactivated bleaching and significantly higher whitening than nonactivated immediate bleaching. Thus, the null hypothesis stating that there is no significant difference between the whitening effect of laser-activated and nonactivated bleaching techniques was rejected.

In this study, tea was the stain of choice, although this may be criticized since intrinsic discoloration of teeth is caused by blood chromogen. 11 However, tea was reported to produce an intrinsic stain that is consistent with the clinically observed tooth discoloration. 12

Several electronic devices have been used for *in vitro* and *in vivo* color assessment in dentistry, including spectrophotometers, colorimeters, spectroradiometers, and digital cameras. 13 Easyshade, which is a color-matching device based on spectrophotometer technology, was used in the current study. Easyshade system was reported to be a reliable instrument in both *in vitro* and *in vivo* compared to the other devices, which were more reliable *in vitro* than *in vivo* . 14 To standardize the color measurement method, a special holder was fabricated to guide the spectrophotometer tip to the same location on the crown of the sample.

In this study, the CIEDE2000 formula, which was developed to eliminate the deficiencies in the CIE Lab formula with a (1:1:1) ratio, was used to analyze the color changes of specimens, where *K _L_* = *K _C_* = *K _H_* = 1. The color changes were analyzed based on a threshold of clinical acceptability of color changes at Δ *E*_00_ > 1.8. 15 16

Bleaching of nonvital teeth involves a time-consuming procedure known as the walking bleach technique, which uses hydrogen peroxide (H _2_ O _2_ ) or a combination of sodium perborate (SP) mixed with water or H _2_ O _2_ to release active oxygen. 17 The release of the oxidizing oxygen takes place over a range of 10 days, thereafter, the efficacy of the bleaching agent is significantly reduced and the material has to be replaced. Satisfactory whitening results can be achieved after three to six bleaching cycles. 18 The effectiveness of the walking bleaching technique depends mainly on the bleaching agent's concentration, duration, and number of applications. H _2_ O _2_ walking bleach may have a faster aesthetic outcome, but it may result in undesirable consequences such as cervical resorption and irreversible damage to the dentin and surrounding tissues. Therefore, it is recommended that SP should be mixed with water rather than hydrogen peroxide to minimize root resorption occurrence. 19

During the bleaching process, hydrogen peroxide acts as a strong oxidizing agent that converts to free radicals. These radicals attack and break down the pigment molecules within the tooth, which causes tooth discoloration and eventually whitens the tooth. Acceleration of the whitening effect of the bleaching agents can be achieved by heat (thermocatalytic approach), light, or lasers (photo-oxidation approach). With these activation methods, a rise in the temperature of the bleaching agent occurs, thus, the conversion of hydrogen peroxide to free radicals is accelerated causing the carbon chains of the pigments to detach faster. 20 21

Two bleaching materials were selected in this study; A non-laser-activated 35% hydrogen peroxide internal bleaching gel, and a laser-activated 35% hydrogen peroxide red bleaching gel, with glycerol and potassium nitrate.

The results of our study revealed that the color change achieved with a single application of laser-activated internal bleaching either with or without external bleaching is comparable to that of the conventional 5-day nonactivated walking bleaching technique. These results are in agreement with several previous studies that reported the superior performance of laser-activated bleaching compared to other conventional techniques. Dostalova et al. reported that using a diode laser as an energy source achieved acceptable color change in a shorter time, 22 while Suemori et al. reported a higher color difference after using a 405-nm diode laser with 800 mW/cm ^2^ irradiation, compared to that after halogen lamp irradiation of the bleaching agent. 23

Laser-activation of in-office bleaching has been reported to be more effective than visible light activation. The difference is due to the well-defined monochromatic, single-wavelength laser beam, which reduces the possible side effects of other light sources such as overheating. Several diode laser systems with wavelengths ranging from 790 to 980 nm are used for laser-activated bleaching. Other advantages of the diode laser are its small size, portability, and flexible optic fibers. Moreover, laser-activated bleaching's enhanced efficacy can be attributed to the photochemical effect that is based on photon absorption by specific photoinitiators present inside the bleaching agents, which are adjusted to absorb the wavelength of the light source. 24

In contrast, Lo Giudice et al. reported no significant difference in color change between the laser-activated and light-emitting diode-activated bleaching. This study did not report the operating parameters of the diode laser; therefore, the inferior results of the laser bleaching might be attributed to low-power laser settings. 25

The SEM analysis of the bleached dentin revealed little changes on the dentin surface using nonactivated bleaching, compared to the dentin bleached by laser-activation, which showed substantial surface modification following laser-activated bleaching with complete absence of smear layer, surface irregularities, and open dentinal tubules. These observations cannot be attributed to the direct effect of laser on dentin, as diode lasers with wavelengths of between 800 and 980 nm have limited interaction with water and hydroxyapatite, but rather have high absorption peaks for chromophores such as melanin, hemoglobin, and other pigmented proteins and carbonated hydroxyapatite. Hence, the significant surface modification following laser-activated bleaching could be rather attributed to the ability of the hydrogen peroxide as a whitening agent to penetrate the tooth structure due to its acidic nature, 26 and which may be intensified using laser-activation that led to increased demineralization of the dentin surface, removal of the smear layer, and opening of the dentinal tubules. This potent effect of laser-activated bleaching enhances the penetration of the bleaching materials inside the open dentinal tubules, increases the depth of whitening inside the discolored dentin, and accelerates the whitening process to a significantly shorter time.

### Recommendations and Clinical Applications

The findings of the current study recommend the use of diode laser-activated 35% H _2_ O _2_ for internal bleaching of discolored teeth. The laser-activation protocol consists of 30 seconds application with a power of 2 W in non-contact mode (10 mm away) using 300 um non-initiated tip, and then wait for 7 minutes to avoid heat deposition to the surrounding tissues, followed by second laser application for 30 seconds. The whitening effect of this protocol is similar to that obtained by walking bleaching for 5 days, either with or without laser activation.

### Limitations and Future Research Recommendations

This current study was an *in vitro* study performed on extracted human teeth which do not simulate the oral environment, the results of this study should be supported by clinical trials to confirm the efficacy of the laser activation protocol for internal bleaching. The investigation of the intrapulpal thermal changes during laser application is recommended for future studies.

## Conclusion

Compared to the conventional H _2_ O _2_ walking bleaching technique, the proposed 940 nm diode laser-activated bleaching protocol is much faster and equivalently effective in whitening. The improved whitening efficacy of laser-activated bleaching can be attributed to the potent penetration into the dentin and accelerated photo-oxidation approach.
